# Monitoring Biofilm Formation and Microbial Interactions that May Occur During a *Salmonella* Contamination Incident across the Network of a Water Bottling Plant

**DOI:** 10.3390/microorganisms7080236

**Published:** 2019-08-02

**Authors:** Foteini Karampoula, Agapi I. Doulgeraki, Christos Fotiadis, Anastasia Tampakaki, George-John E. Nychas

**Affiliations:** 1 Department of Food Science and Human Nutrition, Laboratory of Microbiology and Biotechnology of Foods, Agricultural University of Athens (AUA), Iera Odos 75, 11855 Athens, Greece; 2 Institute of Technology of Agricultural Products, Hellenic Agricultural Organization—DEMETER, Sof. Venizelou 1, Lycovrissi, 14123 Attica, Greece; 3 Department of Crop Science, Laboratory of General and Agricultural Microbiology, Agricultural University of Athens (AUA), Iera Odos 75, 11855 Athens, Greece

**Keywords:** *Salmonella*, contamination, bottling plant, bioreporters, microbial interactions

## Abstract

The present study aims to monitor the ability of *Salmonella* to colonize and compete as a member of the mixed species biofilm within key points at a water bottling plant, in case of a contamination incident with this major foodborne pathogen. To achieve this goal, bacterial communities throughout the production line were collected and their identities were investigated by microbial counts and polymerase chain reaction-denaturing gradient gel electrophoresis (PCR-DGGE). These bacterial communities alone or along with constructed *Salmonella* *enterica* serovar Typhimurium (ST) fluorescence-based bioreporters were left to form a biofilm on stainless steel for 6 days at 20 °C. ST bioreporters were constructed by introducing plasmids expressing EYFP (enhanced yellow fluorescent protein) fusions of the genes *csgB*, *csrA*, *sspH2*, and *fliD* into ST 14028S. The bead vortexing-plate counting method was applied for the enumeration of the biofilm population, while the behavior of the bioreporters was evaluated by fluorescence microscopy. From a set of 16 samples that were collected from the plant, species of *Citrobacter*, *Staphylococcus*, *Pseudomonas*, *Bacillus*, and *Exiguobacterium* were identified. The presence of these indigenous bacteria neither inhibited nor enhanced the biofilm formation of ST in mixed bacterial communities (*p* > 0.05). Furthermore, the *csr*A-based bioreporter was shown to be induced in multispecies biofilms with *Citrobacter*. In conclusion, this study enhanced our knowledge of bacterial interactions occurring within a biofilm in a water bottling plant.

## 1. Introduction

The presence of *Salmonella* in the food chain is of great economic significance, as it is the most important, and most recognized, zoonotic pathogen. *Salmonella* is transmitted to humans by the faecal–oral route, whereby contaminated food or water from the intestinal contents of an infected animal are ingested. Indeed, recent information, either on the prevalence of *Salmonella* spp. in natural freshwaters and drinking waters [1,2] or in food [3], does indicate the importance of this pathogen in the food chain. *Salmonella* outbreaks related to food crops have been linked to the contamination of irrigation waters [2]. In 2013, *Salmonella* was associated with a waterborne outbreak in France, where one out of six cases were hospitalized [3]. Although water-borne salmonellosis has been particularly connected with the problematic situations in developing countries, cases were reported earlier in developed counties [2].

The ability of this pathogen to survive and persist in non-host environments, and subsequently transmit to new hosts, could be attributed to the *Salmonella*’s ability to form biofilms on inert surfaces [4]. Indeed, in several studies, the ability of *Salmonella* to easily attach to various food-contact surfaces (such as stainless steel, plastic, and cement) and form biofilms either on biotic or abiotic surfaces [5,6,7,8,9,10] has been shown. It should be stressed, however, that in their natural environment, biofilms constitute predominantly complex multispecies mixtures and do not really resemble the model monospecies (single species) biostructures studied by the majority of laboratories [11].

Variations between planktonic and biofilm cells, as well as between biofilm cells at different stages of biofilm formation, have been recorded [12]. At present, it is also known that the cells are characterized by high heterogeneity in their gene expression levels because of their diverse physiological states within multi- or mono-species biofilms [13]. Thus, it has to be noted that the application of gene expression methods, considering the average gene expression level, may conceal the differences that occur within the subpopulations of the biofilm [12,14]. Over the years, several methods have been proposed and applied in gene expression studies. These methods include microarrays, real time polymerase chain reaction (Real-Time PCR), quantitative Real-Time PCR, RNA sequencing (RNA seq), and serial analysis of gene expression (SAGE) [15,16,17]. Although the application of these techniques has several advantages, several drawbacks are also present, including cost, specificity, difficulties to perform the experiments, analysis, and reliability [16]. Bacterial bioreporters could be an alternative method for monitoring environmental concerns in water, air, soil, and food samples, which attests to the simplicity of their use. However, the signals produced by the cells are not always easy to interpret [18].

The present work aims to assess whether there is a high incidence for *Salmonella* biofilm formation if the bottling plant is contaminated with this major foodborne pathogen. Thus, a case scenario was designed to study the behavior of the *Salmonella enterica* serovar Typhimurium throughout the different steps across a production line in the water bottling industry. To achieve this, the following objectives have been set: (a) the collection of water samples from different steps of the production line (water source, water distribution network, and final product) into the local bottling plant; (b) the molecular identification of mixed communities recovered from the samples; (c) the assessment of biofilm formation ability and microbial interactions of *Salmonella* and the aforementioned mixed communities in vitro; and (d) investigation of the behavior of *Salmonella* under conditions partially simulating (in vitro) an industrial environment by fluorescence-based bioreporters in mixed biofilms.

## 2. Materials and Methods

### 2.1. Strains, Media, and DNA Manipulations

*Escherichia coli* strains DH5alpha and DB3.1 (Invitrogen, Carlsbad, CA, USA) were used for general cloning and Gateway technology (Invitrogen) manipulations, respectively. *E. coli* and *Salmonella enterica* serovar Typhimurium 14028S were routinely grown in a Luria–Bertani (LB) medium at 37 °C. The antibiotics ampicillin (100 µg mL^−1^), kanamycin (50 µg mL^−1^), spectinomycin (50 µg mL^−1^), and chloramphenicol (20 µg mL^−1^) were used (Sigma–Aldrich, St. Louis, MO, USA).

Following the manufacturer’s instructions, we used the GenElute Bacterial Genomic DNA Kit (Sigma) to isolate genomic DNA. The Qiagen Spin Miniprep Kit and Clean-up kit were applied for plasmid isolations and DNA enzyme cleanups, respectively. PCR amplifications were performed in a DNA thermocycler (M. J. Research, reno, NV, USA) using GoTaq DNA polymerase (Promega, Madison, Wi, USA) and Q5 High-Fidelity DNA Polymerase (Biolabs, Ipswich, MA, USA) in 50 µL volumes. The primers used are presented in Table 1. Plasmid transfers to *E. coli* and *S. enterica* were accomplished by electroporation (GenePulser; Bio-Rad, Hercules, CA, USA).

### 2.2. Plasmid Constructions

To construct the EYFP (enhanced yellow fluorescent protein) translational fusions used in the present study, DNA fragments containing the promoter and coding regions of the genes *csrA* (STM14_3412), *csgB* (STM14_1309), *sspH2* (STM14_2769), and *fliD* (STM14_2380) were PCR-amplified from the chromosomal DNA of the *Salmonella enterica* serovar Typhimurium 14028S using the corresponding primer pairs listed in Table 1. The primers contained engineered restriction sites (NcoI and XhoI) at their ends for cloning into the corresponding sites of the entry Gateway vector pENTR11 (Invitrogen). The entry clones were recombined into the destination vector pRH008 [22] using LR clonase (Invitrogen) to produce the plasmids pRH008/*csrA*-*eyfp*, pRH008/*csgB*-*eyfp*, pRH008/*sspH2*-*eyfp*, and pRH008/*fliD*-eyfp. The vector pRH0008 is a promoterless Gateway vector that allows the creation of transcriptional or translations fusions to the EYFP (enhanced yellow fluorescent protein). The final destination clones were confirmed by sequencing. Plasmids expressing EYFP fusions of the genes *csgB*, *csrA*, *sspH2*, and *fliD* were introduced into *S. enterica* 14028S by electroporation to produce strains B-418, B-421, B-423, and B-424, respectively.

### 2.3. Water Sample Collection and Sampling

Water samples were collected from the water source (WS), water distribution network (WD), and final product (FP) of a bottling plant. Five to six samples in a total volume of 1500 mL were collected from each sampling point over a period of one month according to the production schedule of the bottling plant. All samples were microbiologically analyzed in the water bottling plant, without been diluted, using the membrane filter technique according to Venieri et al. [23]. In brief, 250 mL of water samples were poured into a filter funnel and drawn through a membrane filter with a pore diameter of 0.45 μm. Bacteria were retained on the surface of the membranes by placing them on the following suitable selective media and incubating them at 37 °C for 48 h: A Pseudomonas selective agar, for the enumeration of *Pseudomonas* spp; an m Endo Agar LES, for total coliforms; the Slanetz and Bartley medium, for enterococci; and a plate count agar, for the enumeration of the heterotrophic plate count (HPC) [24]. After enumeration of the colony forming units, the filter membrane of HPC was held vertically with the forceps into a sterilized tube and was rinsed with 5 mL ¼ strength Ringer’s solution. The membrane was removed, 1 mL glycerol (20% *v*/*v* final concentration of glycerol) was added, and the tube was temporarily stored at −70 °C in the water bottling plant. After collection of all the samples, tubes were transferred to the laboratory under freezing conditions and centrifuged at 5000× *g* for 10 min (4 °C). The supernatant from each tube was removed, and the pellet was resuspended using 2 mL of LB broth with 0.5 mL of added glycerol (20% *v*/*v* final concentration of glycerol). This suspension was stored at −80 °C until further use.

### 2.4. DNA Extraction and Bacterial Identification

The genomic DNA was extracted from each sample and amplified according to Ercolini et al. [25]. In brief, the variable V6-V8 region of the 16S rRNA gene was amplified using the primers U968 (AACGCGAAGAACCTTAC) and L1401 (GCGTGTGTACAAGACCC), giving PCR products of about 450 bp. A GC clamp was added to the forward primer [26]. A PCR reaction was performed in the thermal cycler BioRad ProFlex System and PCR amplification was checked with simple agarose gel electrophoresis in 2% agarose gel at 80 V for 1 h. The gel was then stained with ethidium bromide (0.5 mg mL^−1^) in water before being photographed using a GelDoc system (Biorad).

PCR products were analyzed by denaturing gradient gel electrophoresis (DGGE) using a DCode apparatus (Biorad) [25]. Briefly, samples were loaded on 7% (*w/v*) polyacrylamide gels, containing a 20%–50% urea-formamide denaturing gradient (100% corresponded to 7 M urea and 40% (*w/v*) formamide). The gels were run for 10 min at 50 V, followed by 4 h at 200 V at 60 °C. After the gel was stained with ethidium bromide for 5 to 20 min, it was photographed using a GelDoc system. Differing sequences of DNA (from different bacteria) denatured at different denaturant concentrations resulting in a pattern of bands. Different bands were carefully cut out from the gel and placed in a microfuge tube. At that point, the samples were stored until sequencing.

Bands isolated from the DGGE gel were sent to CeMIA, (Department of Immunology and Histocompatibility, Faculty of Medicine, University of Thessaly, Volos, Greece) and the V6-V8 variable region of the 16S rRNA gene was subjected to sequencing with the primer L1401. Bacterial identification was completed using the BLAST bioinformatics program for sequence searching and comparing. In brief, the BLAST program of GenBank was used to align the sequences and determine their closest known relatives to the partial 16S rRNA gene [27]. The BLAST test was performed against the current Reference RNA sequences (refseq RNA) Database at NCBI (https://www.ncbi.nml.nih.gov/refseq/targetedloci).

### 2.5. Evaluation of Biofilm Forming Ability

Stainless steel (SS) coupons (3 × 1 × 0.1 cm, type AISI-304, Halyvourgiki Inc., Athens, Greece) were the abiotic substrates used for biofilm formation, since this material is frequently used for the manufacture of food processing equipment. Prior to use, the coupons were cleaned according to the procedure described by Giaouris et al. [10]. Following cleaning, the coupons were individually placed in glass test tubes containing 4 mL ¼ strength Ringer’s solution (length 5 cm, diameter 1.5 cm) and autoclaved at 121 °C for 15 min.

During this study, gene expression was evaluated in 6 day biofilms of both monospecies and multispecies *Salmonella*. *S.* Typhimurium. The biofilms were developed in the absence of antibiotics, as in trial experiments it was confirmed that antibiotics did not affect the fluorescence ability of the reporters. Samples collected at the water plant were inoculated in SS coupons and checked for their biofilm forming abilities. Subsequently, each water sample was inoculated in combination with each *S.* Typhimurium reporter (inoculum size ca. 10^6^ CFU mL^−1^). Biofilm development and quantification of the biofilm population by “the bead vortexing method” was performed according to Kostaki et al. [28]. Briefly, all cultures were resuscitated twice in LB broth 37 °C or 20 °C before inoculation. The cells were harvested by centrifugation (5000× *g*, 10 min, 4 °C). The supernatant was removed and the pellet was resuspended in 10 mL ¼ strength Ringer’s solution. Six serial dilutions were prepared in ¼ strength Ringer’s solution. A volume of 500 μL from the 2nd dilution (to give a final population of ca. 10^6^ CFU mL^−1^) was added in the tube containing the coupon. Adhesion was performed by sedimentation for 3 h at 15 °C. Subsequently, the coupon was removed from the tube with the planktonic bacterial suspension and placed in a tube containing LB broth. The coupons were incubated at 20 °C for 6 days without shaking. Two broth changes were realized during each incubation period. For quantification of the biofilm population, the coupon was held vertically with forceps, and each side was rinsed with 5 mL ¼ strength Ringer’s solution to remove planktonic and loosely attached cells. The coupon was then placed into a falcon containing 6 mL ¼ strength Ringer’s solution and 10 glass beads (diameter 3 mm). The falcon was vortexed for 2 min to detach all attached cells. Serial dilutions were prepared in ¼ strength Ringer’s solution and spread on a Tryptic Soy Agar (TSA) (incubation at 20 °C for 24–48 h) and Xylose Lysine Deoxycholate (XLD) (incubation at 37 °C for 24 h). CFUs were counted to determine the cell concentration of the 6 day biofilm.

### 2.6. A Study of Monospecies and Multispecies-Biofilm Formation and the Virulence of Salmonella Typhimurium with Fluorescence Microscopy

After formation of the 6 day biofilm, the coupon was held vertically with forceps, and each side was rinsed with 5 mL ¼ strength Ringer’s solution to remove planktonic and loosely attached cells. The coupon was placed on a microscope slide and then mounted on the stage of the N-400 FL Epi-fluorescence microscope. The 100 W Hg lamp was used as a light source in the fluorescence microscope. The blue excitation filter (excitation wavelength 450–480 nm) was selected, as excitation peaks for GFP and EYFP are close to these wavelengths. The 100X immersion oil objective was used for the specimen’s observation. Each observation started from the left edge of the coupon and then covered the whole length (30 mm) and ended at the right edge of the coupon. This observation method was applied to all coupons to ensure that the sampling area (0.13 mm × 30 mm = 3.9 mm^2^) was the same for each case. Gene expression was studied qualitatively by recording the presence or absence of fluorescent cells. Points in the biofilms where fluorescent cells could be detected (positive results) were photographed using the Pinnacle Studio software, a video editing program, developed by Pinnacle Systems (http://www.pinnaclesys.com).

### 2.7. Data Analysis

All microbiological data were analyzed for statistical significance with analysis of variance (ANOVA). Duncan’s multiple range test was used to determine the significant differences among results at a 95% confidence level.

## 3. Results and Discussion

### 3.1. Water Sample Collection and Bacterial Identification

Α set of 16 samples was collected from the water source (WS), water distribution network (WD), and final products (FN) of a local water bottling plant to monitor the presence of and identify, the bacteria throughout the production line within the plant. In Table 2, the HPC enumerated in different samples and steps are presented. Among all samples tested, total coliforms were enumerated only in WS3 and WD4 (<4 cfu/250 mL and <4 cfu/250 mL, respectively) samples. Total coliforms and especially the genera *Citrobacter*, *Klebsiella*, and *Enterobacter* are present not only in the intestines of mammals and fecal matter, but also in the aquatic environment, soil, and vegetation [29,30]. However, their consideration as good indicators for fecal contamination may be faulty due to their abundance in nature and the harmlessness of many strains [31]. None of the other bacterial indicators for contamination were detected.

**Figure 1:**
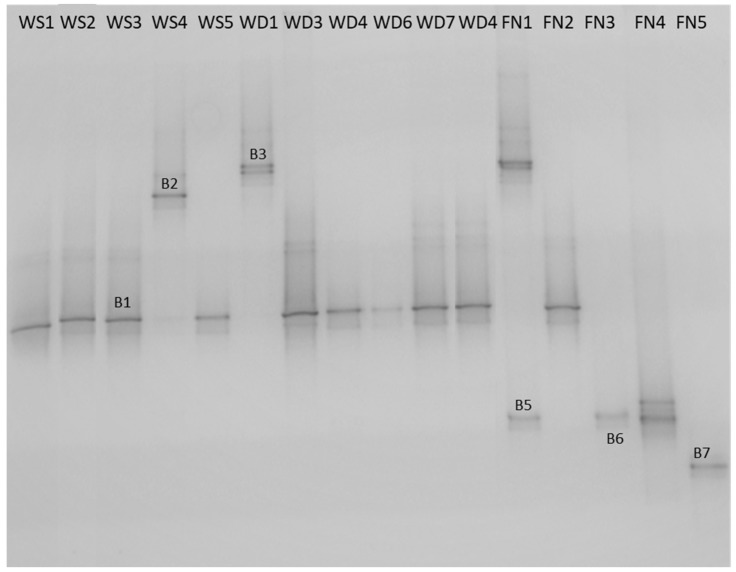
PCR-DGGE profiles of the V6–V8 amplicons of 16S rDNA of mixed communities as recovered from the membrane filter of the collected samples from the water bottling plant. The lane codes represent the number and source of sample; water source (WS); water distribution network (WD); final products (FN). Bands: B1 *Citrobacter* spp., B2 *Staphylococcus* spp., B3 *Staphylococcus* spp., B5 *Pseudomonas* spp., B6 *Bacillus* spp. and B7 *Exiguobacterium* spp.

Regarding the characterization of the mixed communities’ samples, which were subjected to PCR-DGGE analysis, six different DGGE fingerprints (B1, B2, B3, B5, B6, B7) were detected (Figure 1). The DGGE fingerprints were assigned as follows: the B1 to *Citrobacter* spp., the B2 to *Staphylococcus* spp., the B3 to *Staphylococcus* spp., the B5 to *Pseudomonas* spp., the B6 to *Bacillus* spp. and the B7 to *Exiguobacterium* spp. (Table S1). The distribution of these bacteria throughout the three most important stages in the production line into the water bottling plant is presented in Table 2. *Citrobacter* spp. was detected in all three basic stages of the production line (10 of 16 samples). Along with *Citrobacter* spp., *Staphylococcus* spp. were the second most common bacteria detected in the bottling plant (4 of 16 samples). *Bacillus* spp. were detected in two samples, which were both final products. Species of *Citrobacter* genus can cause opportunistic infections in humans (e.g., *Citrobacter freundii*) [32], so the persistence of *Citrobacter* throughout the bottling plant may be alarming. However, it is necessary to identify the exact species and, subsequently, conduct attentive sampling, microbiological tests, and virulence tests to confirm the problem’s occurrence, determine its origin, and make appropriate correction actions. The occurrence of *S. aureus* in drinking water may pose an important health hazard, especially if a high cell population, antibiotic resistance, and/or enterotoxin production ability coincide [33]. Furthermore, the occurrence of *Exiguobacterium* spp. and *Pseudomonas* spp. in drinking water has also been reported previously [31,34,35,36].

### 3.2. Biofilm Forming Ability and Microbial Interactions of Bacteria Recovered from the Bottling Plant and Salmonella on Stainless Steel Surfaces

**Figure 2:**
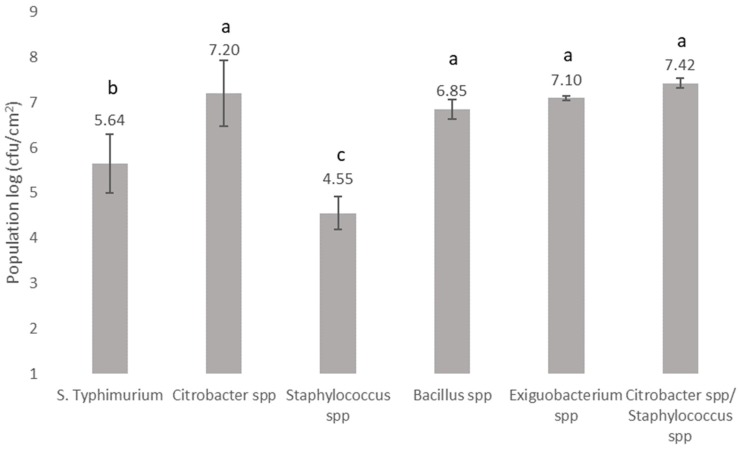
The cell density (log cfu/cm^2^) of biofilms developed at 20 °C for a total period of 6 days by *Salmonella* Typhimurium and samples recovered as bulk cells from the bottling plant. The identification was based on the BLAST analysis of bands sequences isolated from the DGGE gel. Bars represent means ± standard deviations. The different letters (a,b,c) indicate differences in the biofilm formation capabilities of different species at a probability level of 95%.

**Figure 3:**
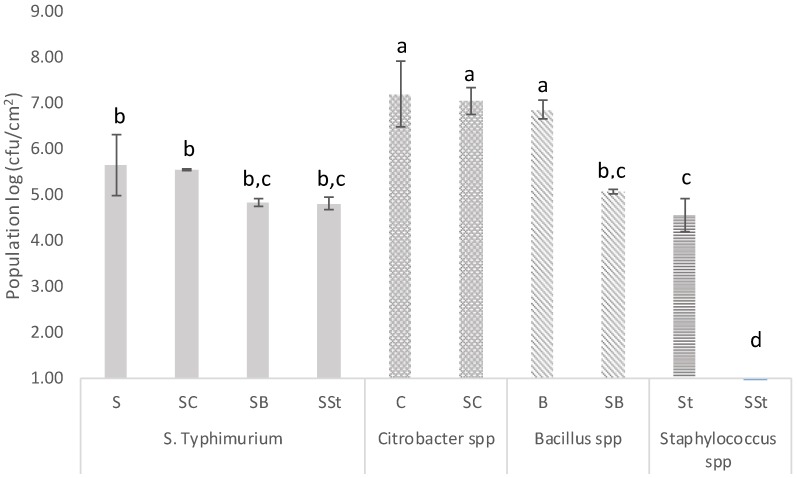
The cell densities (log cfu/cm^2^) of *Salmonella enterica* serovar Typhimurium (S), *Citrobacter* spp. (C), *Bacillus* spp. (B), and *Staphylococcus* spp. (St) single (single letter e.g S) and mixed (double letter e.g SSt) biofilms at 20 °C, for a total period of 6 days, with samples recovered as bulk cells from the bottling plant. The identification was based on the BLAST analysis of the band sequences isolated from the DGGE gel. Bars represent means ± standard deviations. The different letters (a,b,c,d) shown above indicate differences in the biofilm formation capability of different combinations (single and mixed communities) at a probability level of 95%.

This study aims to assess whether there is a high incidence of the biofilm formation if a bottling plant is contaminated with *Salmonella*. Thus, the biofilm formation ability of *S*. Typhimurium in the presence of bacteria recovered from the bottling plant in a simulated environment was monitored. It has been shown that it is not abnormal to detect a *Salmonella* contamination in different stages of the production line of bottling water. Indeed, *Salmonella* has been detected in natural freshwaters and drinking waters [1,2], as well as in food [3]. Moreover, the ability of *S. enterica* [5,7,37,38], *Staphylococcus* spp. [39,40], *Bacillus* spp. [41,42], and *Citrobacter* spp. [43] to form biofilms on stainless steel surfaces has been previously reported. In this study, the potential of the recovered communities from the water bottling plant, along with the tested pathogen, to form a biofilm on stainless steel surfaces was examined. According to the obtained results, samples consisting of *Citrobacter* spp., *Exiguobacterium* spp., or *Bacillus* spp. displayed the greatest biofilm populations (*p* > 0.05) followed by *S.* Typhimurium and samples that consisted of *Staphylococcus* spp. (*p* < 0.05) (Figure 2). For the samples where *Citrobacter* spp. and *Staphylococcus* spp. were both detected by PCR-DGGE analysis, the biofilm population reached 7.42 log cfu/cm^2^, similar to the population enumerated for the sample consisting of *Citrobacter* spp. (*p* > 0.05). Furthermore, the population of a multispecies biofilm consisting of bacteria recovered from the bottling plant as bulk cells, as well as the foodborne pathogen *S.* Typhimurium, is shown in Figure 3. In multispecies biofilms of *S.* Typhimurium and *Citrobacter* spp., the latter species dominated the bacterial biofilm community. It has been reported previously that *Citrobacter* spp. is a strong competitor in mixed biofilms with *E. coli* and *L. monocytogenes* [44,45]. However, the individual cell densities of both bacteria in the mixed biofilm were almost the same as in single-species biofilms (*p* > 0.05). This result implies that competitive interactions may not develop between these particular species, as both of them did not face a reduction in biomass when they coexisted. Furthermore, in multispecies biofilms of *S.* Typhimurium with *Bacillus* spp., cell densities of the two species were almost the same but lower compared to single-species biofilms of *Bacillus* spp. (*p* < 0.05). Indeed, in the case of the sample consisting of *Bacillus* spp., a 2 log reduction was observed, suggesting that competitive interactions may occur in this case. The cell densities of samples consisting of *Staphylococcus* spp. in multispecies biofilms with *S.* Typhimurium were below the detection limit of the plate count method (1 log cfu/cm^2^). In recent years, researchers have focused on the influence of the complexity and interactions in multispecies biofilms [46]. However, the inhibitory impact of *S. enterica* on *Staphylococcus* spp. growth in mixed biofilms has not been previously reported.

The present study showed that the bacterial species recovered from the bottling plant were capable of forming biofilms on stainless steel, and, hence, they were included in studies examining the multispecies biofilm development on such surfaces. Multispecies biofilms consisting of *S.* Typhimurium and bacterial species recovered from a bottling plant formed on stainless steel and competitive interactions were recorded between *Salmonella* and *Bacillus* or *Staphylococcus* under the experimental conditions tested (*p* < 0.05). However, the commonly detected species *Staphylococcus* and the rarely detected species *Bacillus* were unable to influence the biofilm formation capability of *S.* Typhimurium, indicating that the presence and effect of other species on biofilm formation is species specific and should be considered. These observations support our concern that biomass losses and gains are often not enough to characterize interactions as cooperative or competitive, as many factors should be taken into consideration when exploring complex systems. For example, one species may face a reduction in biomass by joining a multispecies community, but it may gain increased protection from various stresses or an expanded niche, resulting in an overall fitness gain [47].

### 3.3. Monitoring the Biofilm Formation and Virulence of Salmonella in Monospecies and Multispecies Communities by Fluorescent-Based Bioreporters

To further investigate the biofilm formation and microbial interactions of *Salmonella* within a mixed bacterial community by means of fluorescence microscopy, whole-cell bioreporters of *Salmonella* were used. Genetically engineered *S.* Τyphimurium bioreporter strains were constructed, based on genetic fusions of the green fluorescent protein with promoters from several genes that are highly associated with biofilm formation and virulence in *Salmonella*. Specifically, *S*. Typhimurium bioreporters expressing EYFP fusions of the genes *csgB*, *csrA*, *sspH2*, and *fliD* were constructed (Table 1). Biofilm formation and virulence were observed by fluorescence microscopy, and gene expression was evaluated by recording the fluorescence signal from individual *Salmonella* cells expressing fluorescent bioreporters.

**Figure 4:**
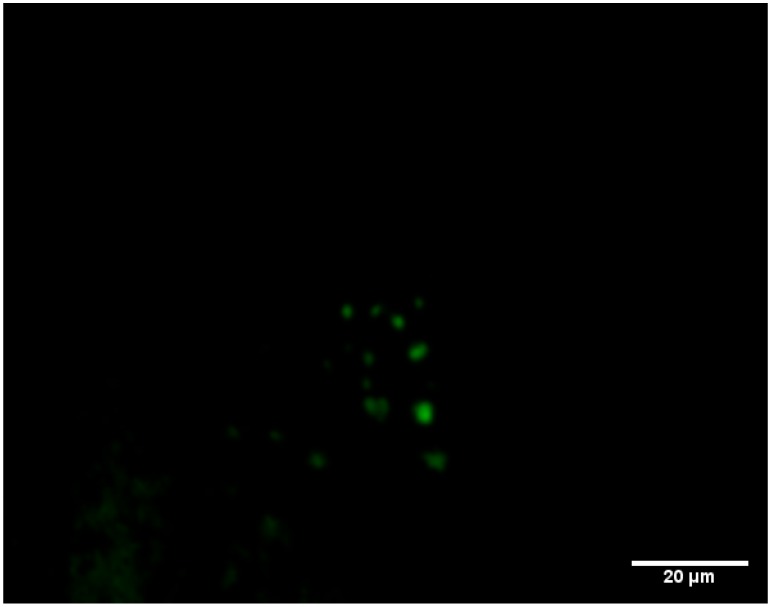

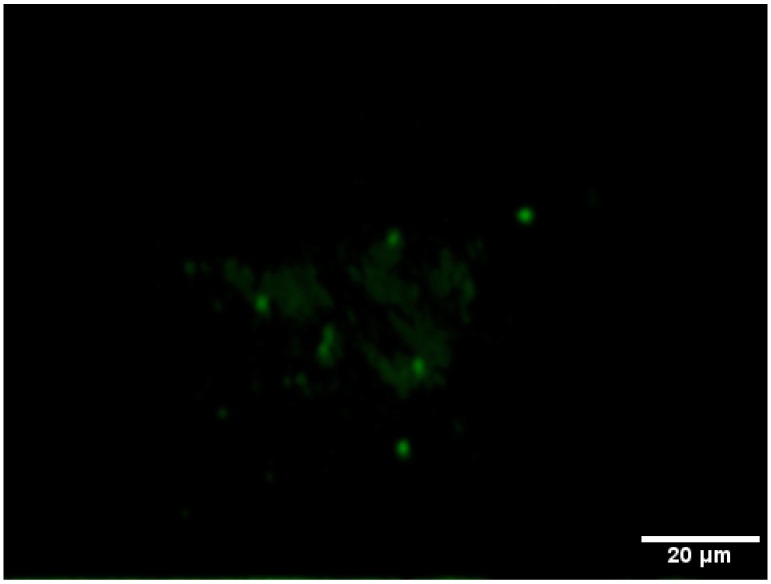
Fluorescence microscopy images of biofilm cells on a stainless steel coupon of *Salmonella enterica* serovar Typhimurium in multispecies biofilms with the *Citrobacter* spp. recovered as bulk cells from the bottling plant. Green fluorescent cells indicate the expression of the csrA EYFP (enhanced yellow fluorescent protein) reporter in *S*. Typhimurium. Two photos represent different observation areas on stainless steel coupons.

In general, the spatial distribution of weak fluorescent cells was observed for the EYFP reporters *csgB*, *sspH2*, and *fliD*, in both single-species and multispecies *S*. Typhimurium 6 day biofilms, but this was not the case for the B-421 bioreporter expressing EYFP under the control of the *csrA* promoter. Interestingly, in the case of B-421, there were many fluorescent cells in multispecies 6 d biofilms, with samples consisting of *Citrobacter* spp. (Figure 4). In addition, there were no fluorescent cells in monospecies biofilms or multispecies biofilms of *Salmonella* with other microorganisms identified in the present study. The bioreporter strain B-421 possessed an important advantage, as the fluorescence signal was localized in a small region into the cell resulting in a more intense fluorescence compared to other EYFP reporters. B-421 harbors the plasmid with the *csrA* gene promoter regulating EYFP expression. The global carbon storage regulatory system (Csr) exploits a family of RNA binding proteins (CsrA (RsmA) proteins, which are the principal components of this post-transcriptional system. In specific, CsrA has been reported to contribute as a control mechanism to many cellular functions and virulence, including motility, quorum sensing, carbon metabolism, interaction with hosts, and biofilm production has been reported [48,49,50]. Biofilm formation has been shown to repress biofilm formation post-transcriptionally by direct or indirect effects on EPS production, indirect effects by inducing motility, and a direct effect on levels of C-di-GMP—a principal molecule for the activation of biofilm dispersal by switching the cell mode from planktonic to sessile [51].

The present study showed that the *csrA* promoter is induced in multispecies biofilms of *Salmonella* with *Citrobacter* spp. It has been reported that the switching of *Salmonella*’s cell mode from sessile to motile is strongly controlled by CsrA at multiple levels [52]. More specific, the biofilm dispersal seems to be activated by CsrA, as viable planktonic cells are released from the biofilm after the induction of this specific gene. It could be presumed that biofilm dispersion is likely activated early in multispecies biofilms with *Citrobacter* spp. because of its high cell densities, which cause unfavorable conditions, such as starvation. The contribution of CsrA to the regulation of *Salmonella* virulence genes is also known. Therefore, the *Citrobacte*r-dependent induction of *csrA* in *Salmonella* highlights the importance of preventing biofilm formation by this foodborne pathogen and bacteria commonly found in the food industry. The *csrA*-based bioreporter was shown to be suitable for biofilm detection in mixed populations containing *Citrobacter* and *S.* Typhimurium. Taking into account that interspecies interactions may alter the phenotype, physiology, metabolism, and gene expression of a pathogenic bacterium like *S.* Typhimurium, analogous case studies should be tested in more detail. Moreover, *csrA* could constitute a valuable tool to monitor the biofilm formation of *Salmonella* in multispecies biofilm containing *Citrobacter* spp. These results suggest that genes other than *csrA* may be used as bioreporters for monitoring biofilm formation in mixed populations of *Salmonella* with bacteria other than *Citrobacter* spp. Future research is needed to determine whether other bacterial species are capable of inducing the biofilm formation of *Salmonella* in multispecies biofilms. Moreover, other biofilm-related genes should be tested for their ability to detect biofilm formation in mixed populations containing a wider range of bacterial species.

## 4. Conclusions

In conclusion, this study enhances our knowledge of microbiota presented along the production line within a water bottling plant. It was shown that total coliforms are relatively abundant and can readily form biofilms on stainless steel surfaces, which allows an in vitro study of multi-species biofilm formation. Additionally, these results provide a suitable experimental platform to study bacterial species interactions, the mechanisms of which underlie these interactions. These results could also enable the development of control mechanisms of bacterial biofilms on industrial surfaces. Furthermore, the ability of *S*. Typhimurium to form a biofilm (highlighted in this study) emphasizes the importance of controlling the environment of the bottling plant so that pathogens cannot enter the production environment. Lastly, the exploitation of suitable whole cell bioreporters will enable researchers to detect and understand biofilm formation in mixed bacterial populations on both food and industrial surfaces.

## Figures and Tables

**Table 1:** The oligonucleotide and polymerase chain reaction (PCR) conditions used in the present study.

Gene	Gene Function	Primer Name	Primer Sequence (5′ to 3′) ^a^	Restriction Sites	Amplified Region ^b^	Reference
*csrA*	General regulator^:^ motility, biofilm formation, virulence [19]	csrA-F1	TTAAACCATGGGAATTGATTGGGATGGTCGCTC	NcoI	−389…+183	This study
		csrA-R1	GTAACTCGAGTGCTGGGATTTTTCAGCCTGG	XhoI		This study
*csgB*	Nucleator in the assembly of curli (coiled surface structures) on the cell surface [19]	csgB-F1	ATCCCCATGGCGGCATAACACACACCAAC	NcoI	−374…+156	This study
		csgB-R1	CTATTACTCGAGCCGACTTGACCAATAATGGC	XhoI		This study
*sspH2*	virulence factor [20]	sspH2-F1	GCGTTCCATGGTTGCCTGATACGGATGAAAACCG	NcoI	−374…+159	This study
		sspH2-R1	CAAATCCTCGAGATGCACTCCAGCGCTTCAGTC	XhoI		This study
*fliD*	Motility regulator [21]	fliD-F1	GCATACCATGGAACAGTTGCAGCCGTATCGCTG	NcoI	−831…+48	This study
		fliD-R1	TTTTCCTCGAGCCATAGGCGGTTAGCTTTGCCG	XhoI		This study

^a^ The underlines denote the restriction enzymes sites. ^b^ The numbers denote the positions of the amplified regions, containing promoter and coding sequences, relative to the translational start site of the corresponding gene.

**Table 2:** Distribution of species obtained from the heterotrophic plate count (HPC) medium according to PCR-DGGE profiling throughout production line within the water bottling plant.

Microorganism	Source/Sample															
	Water Source					Water Distribution Network						Final Products				
	WS1	WS2	WS3	WS4	WS5	WD1	WD3	WD4	WD6	WD7	WD10	FN1	FN2	FN3	FN4	FN5
HPC (cfu/mL) *	4	5	3	29	5	12	7	9	23	26	4	16	15	30	1	3
*Citrobacter* spp. **	+	+	+		+		+	+	+	+	+		+			
*Staphylococcus* spp. **				+												
*Staphylococcus* spp. **				+		+					+	+				
*Pseudomonas* spp. **												+			+	
*Bacillus* spp. **														+	+	
*Exiguobacterium* spp. **																+

* Heterotrophic plate counts (HPC) were detected by membrane filter method and the results were provided by the water bottling plant. ** The identification was based on a BLAST analysis of band sequences isolated from denaturing gradient gel electrophoresis (DGGE) gel. WS, water source; WD, water distribution; FN, final products.

## Supplementary Materials

The following are available online at https://www.mdpi.com/2076-2607/7/8/236/s1. Table S1. Identity of isolated bands from PCR-DGGE fingerprints of mixed communities as recovered from membrane filter of collected samples from the water bottling plant after sequencing of the variable V6-V8 region of 16S rRNA genes.
