# Supplementary material for: Monitoring Biofilm Formation and Microbial Interactions that May Occur During a Salmonella Contamination Incident across the Network of a Water Bottling Plant
Source: Microorganisms. 2019 Aug 2;7(8):236. doi: 10.3390/microorganisms7080236 (PMC6723698; doi:10.3390/microorganisms7080236)
Supplement: Supplementary file 1 [file microorganisms-07-00236-s001.pdf]

Table S1. Identity of isolated bands from PCR-DGGE fingerprints of mixed communities as recovered from membrane filter of collected samples from the water bottling plant after sequencing of the variable V6-V8 region of 16S rRNA genes

Band[1]	Closest relative	Identity (%)	GenBank accession no. of closest relative
B1	*Citrobacter* spp.	99	KM187140
B2	*Staphylococcus* spp.	100	MG162677
B3	*Staphylococcus* spp.	99	MK942713
B4	*Pseudomonas* spp.	100	MF144455
B5	*Bacillus* spp.	97	MK660034
B6	*Exiguobacterium* spp.	100	KC160692

[1] Bands are indicated in Figure 1

© 2019 by the authors. Licensee MDPI, Basel, Switzerland. This article is an open access article distributed under the terms and conditions of the Creative Commons Attribution (CC BY) license (http://creativecommons.org/licenses/by/4.0/).
